# Expanding the phenotypic and immunological landscape of Alazami syndrome: Evidence from seven new patients with *LARP7* gene variants

**DOI:** 10.1007/s00431-026-06801-0

**Published:** 2026-03-11

**Authors:** Wessam Sharaf-Eldin, Raghda M. Ghorab, Karima Rafat, Heba Mahmoud, Nehal Hassib, Abdelaziz Alahlafi, Reza Maroofian, Joseph G. Gleeson, Mona Essawi, Maha S. Zaki

**Affiliations:** 1 Depatment of Medical Molecular Genetics, Human Genetics and Genome Research Institute, National Research Centre, Cairo, Egypt; 2 Immunogenetics Department, Human Genetics and Genome Research Institute, National Research Centre, Cairo, Egypt; 3 Department of Clinical Genetics, Human Genetics and Genome Research Institute, National Research Centre, Cairo, 12311 Egypt; 4 Orodental Genetics Department, Human Genetics and Genome Research Institute, National Research Centre, Cairo, Egypt; 5 Dental Consultant, dental clinics, New Giza University, Giza, Egypt; 6 Omar Al-Mukhtar University, Al-Beida City, Libya; 7 Department of Neuromuscular Disease, UCL Queen Square Institute of Neurology and the National Hospital for Neurology and Neurosurgery, London, UK; 8 Department of Neurosciences, University of California, San Diego, La Jolla, CA 92093 USA; 9 Rady Children’s Institute for Genomic Medicine, San Diego, La Jolla, CA 92093 USA; 10 Medical Genetics Department, Armed Forces College of Medicine, Cairo, Egypt

**Keywords:** Alazami syndrome, Neurodevelopmental disorder, LARP7, Immunodeficiency

## Abstract

Alazami syndrome is a neurodevelopmental disorder characterized by postnatal growth retardation, moderate to severe intellectual disability, and facial dysmorphology. It is caused by biallelic variants in the transcriptional regulator La ribonucleoprotein 7 (*LARP7*), where frameshift variants accounted for the majority of cases. The current study presents 7 new patients, including 3 males and 4 females from 3 unrelated families. Careful and thorough clinical examination identified novel oro-dental disease abnormalities, including a prominent premaxilla and enamel defects. The detected variants (c.1113_1116del, c.997 + 2T > C and c.518T > C) were not reported in the previous studies. The substitution c.518T > C represented the second missense variant to be identified in patients with Alazami syndrome. Male patients from the three families fulfilled ≥ 2 clinical warning signs of primary immunodeficiency. Lymphocyte subset counts and immunoglobulin levels were estimated in patients from two families. The values were within reference ranges, with only minor non-significant alterations in cytotoxic T-cell counts. A functional assay of B lymphocyte response was performed in one family, demonstrating impaired Streptococcus pneumoniae IgG antibody production following Pneumovax vaccination in the male patient, while his female sibling mounted an adequate response. In conclusion, the disease has a wide range of symptoms, which vary greatly among the affected patients. Our study expanded the clinical and molecular spectrum of the disorder and highlighted immunodeficiency as an underrecognized disease feature, potentially with a male sex predilection.

**Supplementary information:**

The online version contains supplementary material available at 10.1007/s00431-026-06801-0.

## Introduction

Alazami syndrome (OMIM: 615,071) is a rare autosomal recessive neurodevelopmental disorder originating due to pathogenic variants in the *LARP7* gene on chromosome 4q25. Gene variants were first suggested as a cause of intellectual disability in 2011 [1]. However, the disease nomenclature has come after its full delineation by Alazami and his colleagues in 2012 [2]. The cardinal disease characteristics are short stature, intellectual disability, and facial dysmorphology [2, 3]. Other symptoms involving microcephaly, speech impairment, seizures, autistic features, stereotypical hand movements, brain malformations, congenital heart defects, ocular anomalies, skeletal deformities, skin manifestations, behavioral difficulties, and genito-renal anomalies have been reported in a significant proportion of cases [3–5].

Members of the La-related protein (LARP) family mediate various functions in RNA metabolism [6]. All LARPs contain an N-terminal La-motif (LaM), and an adjacent RNA recognition motif (RRM1), together known as La module, which enable LARPs to contact their RNA substrates [7]. Unlike most proteins of the LARP family, *LARP7* contains a second RRM, referred to as RRM2 [8]. *LARP7* is almost expressed in all body tissues with higher prevalence in the brain [9]. It acts as a chaperone protein, where it interacts with the 7SK RNA promoting its stability, and serving as a scaffold for the binding of other proteins forming the 7SK snRNP complex. This complex inhibits the positive transcription elongation factor b (P‐TEFb). P-TEFB is a cyclin-dependent kinase that is essential for the activation of RNA polymerase II and the initiation of transcription. Thus, the 7SK snRNP complex acts as a transcriptional regulator by inhibiting phosphorylation and transcriptional elongation of RNA polymerase II [6].

Management of Alazami syndrome includes multidisciplinary procedures depending on the clinical manifestations, with lifelong follow-up. Life expectancy is currently unknown. However, patients can reach early adulthood [10]. The majority of cases require different degrees of help during their daily activities, where the level of autonomy relies basically on the extent of intellectual disability, lingual delay, and motor handicap. Speech therapy, educational guidance, and neuropsychiatric assistance seem effective [11]. The current study presents 7 new patients from Egypt (*n* = 4) and Libya 3 (*n* = 3) highlighting novel or overlooked disease features.

## Subjects and methods

### Clinical investigations

Patients were recruited from the Neurogenetics clinic at the Medical Research Centre of Excellence (MRCE), National Research Centre (NRC). A complete medical history was obtained, including three-generation pedigree construction, family history, and prenatal, natal, and postnatal histories. Basic anthropometric measurements and a detailed physical examination of all body systems were performed. Additionally, a comprehensive oro-dental examination was done to document the intraoral features. Investigations, including brain neuroimaging, electroencephalogram (EEG), echocardiography, metabolic work up and fundus examination, were also carried out. Informed consent was obtained from patients’ guardians prior to blood sampling and for the publication of their photographs.

### Immunological evaluation

Immunological evaluation included assessment of the ten warning signs of primary immunodeficiency, with suspicion raised when ≥ 2 signs were present [12]. Lymphocyte subset immunophenotyping was performed by flow cytometry (BD Accuri™ C6 Cytometer, USA) on lysed whole blood using standard monoclonal antibodies (CD3, CD4, CD8, CD16, and CD19). For lymphocyte subset analysis, initial gating was performed on CD45⁺ leukocytes using side scatter properties, followed by identification of T cells (CD3⁺), B cells (CD19⁺) and NK cells (CD16⁺). CD4⁺ and CD8⁺ T-cell subsets were subsequently defined within the CD3⁺ T-cell gate. Serum immunoglobulin (IgG, IgA, IgM) levels were measured by immunoturbidimetry. Functional B-cell responses were assessed using the 23-valent polysaccharide vaccine Pneumovax, with serological testing for pneumococcal IgG antibodies by ELISA performed before and one month after vaccination.

### Molecular analysis

Exome sequencing (ES) was performed on genomic DNA extracted from peripheral blood leukocytes of the probands using PAXgene® Blood DNA Kit (Qiagen, Hilden, Germany). Approximately 37.5 Mb of the genome, corresponding to > 99% of the Consensus Coding Sequence was enriched from fragmented DNA using TruSeq Exome Library Prep Kit (Illumina, San Diego, CA, USA). The libraries generated were run on the Illumina NovaSeq 6000 platform (Illumina) using S1, S2, or S4 Reagent Kits (average read depth > 100x). The raw data were demultiplexed to link molecular barcodes with the sample identifiers, followed by adaptor trimming. The reads were then mapped to the human genome reference (GRCh37) and duplicated reads were marked before variant calling and annotation. The bioinformatics procedure includes the detection of germline SNVs, small insertions or deletions, and copy number variations. Pathogenicity of variants was assessed based on the American College of Medical Genetics (ACMG) guidelines [13]. Structural effect of the missense variant was predicted by Hope (https://www3.cmbi.umcn.nl/hope/) [14]. Segregation analysis of the identified variants was carried out in other family members by Sanger sequencing using the Applied Biosystems 3500 genetic analyzer (Thermo Fisher Scientific, Waltham, MA, USA). GeneMANIA was used to explore potential functional and physical interactions between *LARP7* and X-linked immune-related genes [15].

## Results

### Clinical findings

**Fig. 1:**
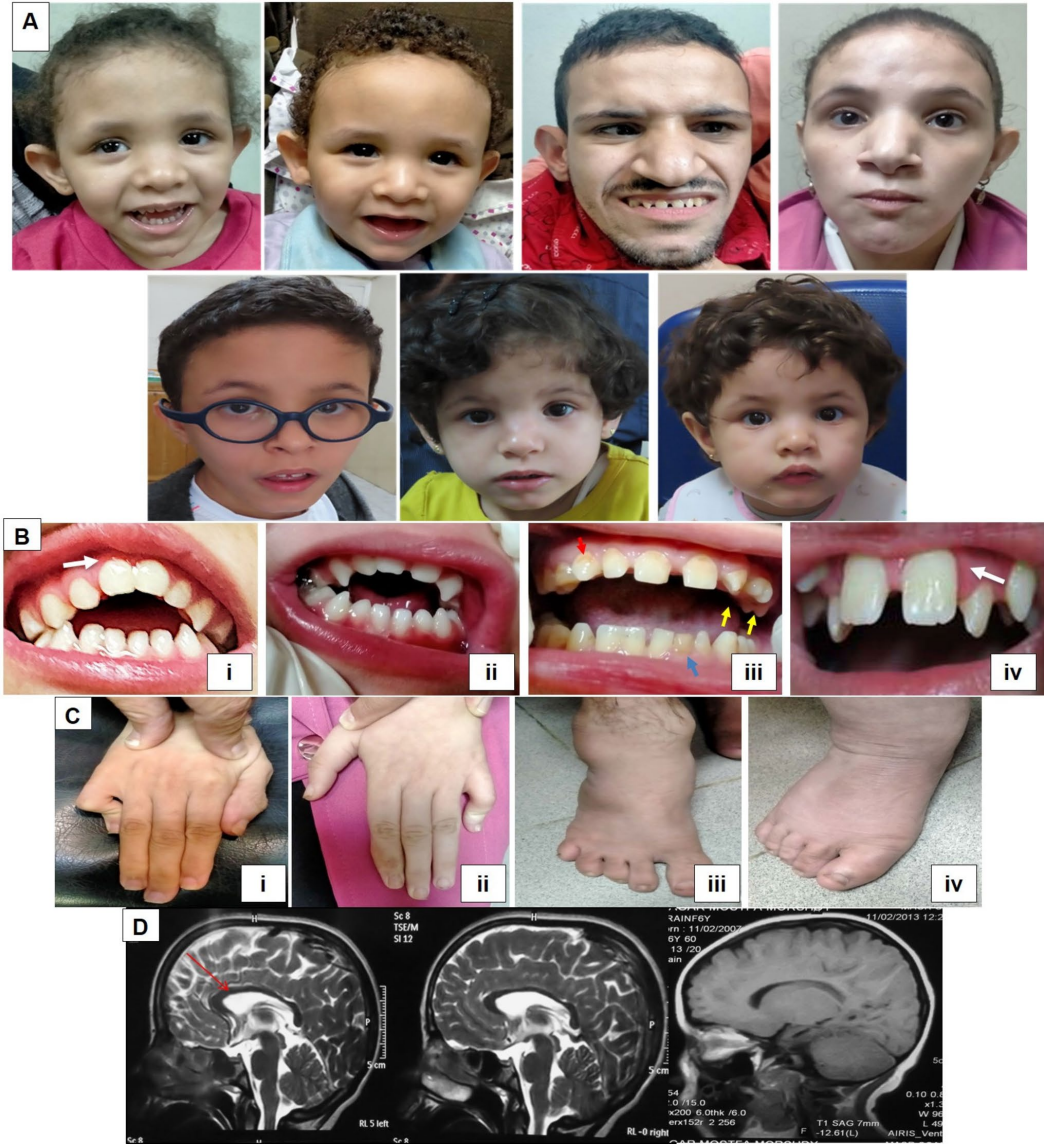
**A**) Facial dysmorphic features of the enrolled Subjects (P1 to P7). **B**) Intraoral photos showing anterior open bite, pointed canines and microdontia in P1 (i), prominent premaxilla (white arrow) and anterior open bite in P2 (ii), widely spaced teeth in upper jaw, enamel hypoplasia (red arrow), rotated dentitions (yellow arrows) and retained deciduous teeth (blue arrow) in P3 (iii) and widely spaced teeth, malposed dentition and prominent premaxilla (white arrow) in P5 (iv). **C**) skeletal abnormalities including arachnodactyly (i), clinodactyly of the 5th finger (ii), abnormal flat feet (iii) and long and broad toes (iv). **D**) Brain MRI indicating thin corpus callosum (red arrow)

The study included 7 patients (3 males and 4 females) from 3 consanguineous families with ages ranging from 2 to 15 years **(**Table 1**, **Supplementary Fig. 1). Anthropometric measurements showed short stature in all subjects. Low weight was present in all individuals except Patient 4, and microcephaly was observed in only four cases. All patients achieved ambulation, although six exhibited motor delays. They all had an intellectual disability with severe speech impairment ranging from complete absence (*n* = 1) to limited ability for only letters (*n* = 1) or a few words (*n* = 5). Certain facial dysmorphic features were observed in all patients, including midface hypoplasia, high forehead, prominent pointed chin, deep-seated eyes, low-set ears, broad nasal root, and hypoplastic alae nasi **(**Fig. 1A**)**. The most frequent oro-dental abnormalities were prominent premaxilla (*n* = 7), thin upper lip (*n* = 7), high arched palate (*n* = 6), anterior open bite (*n* = 5), and enamel hypoplasia/hypocalcification (*n* = 5) **(**Fig. 1B**)**. Behavioral changes such as autistic features (*n* = 6), nervousness (*n* = 4) and hyperactivity (*n* = 3) were common among the enrolled subjects. Neurological examination identified axial hypotonia and mild hypertonia in patients of families 1 and 2, respectively. Reflexes were normal in all patients, except for patient 3 (P3), who exhibited brisk reflexes. Skeletal abnormalities were noted in 6 patients in the form of arachnodactyly (*n* = 5), clinodactyly (*n* = 2), clasped thumb (*n* = 2), and talipes (*n* = 1) **(**Fig. 1C**)**. Cardiac and chest examinations were normal in all subjects. Neuroimaging showed no pathognomonic findings except for a thin corpus callosum in all patients **(**Fig. 1D**)**.

**Table 1:** Clinical and molecular findings of the enrolled subjects

Family #		1		2		3		
Patient #		P1	P2	P3	P4	P5	P6	P7
Sex		F	M	M	F	M	F	F
Age		3y5m	1y10m	15y	11y	11y	3y9m	2y9m
Consanguinity		+	+	+	+	+	+	+
Family history		+	+	+	+	+	+	+
Perinatal history		-	-	-	-	-	-	-
Onset		6 m	6 m	1y	1y	1y	1y	1y
Weight (SD)		9 (−4.1)	6 (−5.7)	28 (−4.8)	43 (−1.7)	25 (−4.09)	13 (−3.8)	12.5 (−3.7)
Height (SD)		88 (−2.9)	80 (−3.06)	130 (−6.4)	131 (−4.8)	136 (−4.2)	100 (−4.9)	98 (−4.3)
Head circumference (SD)		44 (−3.5)	43.5 (−3.67)	50 (−3.4)	51 (−3.1)	52.5 (−1.67)	49 (−2.12)	50 (−1.1)
Microcephaly		+	+	+	+	-	-	-
Low weight		+	+	+	+	+	+	+
Short stature		+	+	+	+	+	+	+
Face shape		long	long	triangular	long	triangular	long	round
Midface hypoplasia		+	+	+	+	+	+	_
Forehead		high flat	high prominent	high prominent	high prominent	high flat	high prominent	high prominent
Prominent pointed chin		+	+	+	+	+	+	+
Dimpled chin		-	-	+	+	+	+	+
Deep-seated eyes		+	+	+	+	+	+	+
Sparse lateral eyebrows		+	+	_	_	_	_	_
Upward slanting palpebral fissures		_	_	_	+	_	_	_
Broad nasal root		+	+	+	+	+	+	+
Broad nasal bridge		_	_	+	+	_	_	_
Bulbous nasal tip		+	+	_	_	+	+	+
Hypoplastic alae nasi		+	+	+	+	+	+	+
Broad prominent nose barrel with prominent columella		_	_	+	+	_	_	_
Low-set ears		+	+	+	+	+	+	+
Short Philtrum		+	+	+	+	_	+	+
Long & broad philtrum		_	_	_	_	+	_	_
Macrostomia		+	_	+	_	_	_	_
Palate		high arched	high arched	shallow	high arched	high arched	high arched	high arched
Anterior open bite		+	+	_	+	_	+	+
Thin upper lip		+	+	+	+	+	+	+
Everted lower lip		_	_	_	_	+	+	+
Prominent premaxilla		+	+	+	+	+	+	+
Retrognathia		_	_	_	_	_	_	+
Wide overjet		_	_	+	_	_	_	_
Macroglossia		_	_	+	_	_	_	_
Microdontia		_	+	_	_	_	_	_
Pointed canines		_	+	_	_	_	_	_
Retained dentition		_	_	+	_	_	_	_
Malposed/rotated dentition		_	_	+	_	+	_	_
Widely-spaced teeth		_	_	+	+	+	_	_
Delayed eruption		_	_	+	_	_	_	
Enamel hypoplasia/hypocalcification		_	+	+	+	+	_	+
Median grooved tongue		_	_	_	+	+	_	_
Delayed motor milestones		+	+	+	+	+	+	_
Age at independent walking		3y4m	2y	2y	3y	2y	2y	1y4m
Intellectual disability (IQ)		severe (33)	moderate (45)	moderate (41)	moderate (43)	severe (34)	moderate (49)	moderate (48)
Speech		letters	few words	few words	few words	absent	few words	few words
Seizures		-	-	-	-	-	-	-
Neurological evaluation	Tones	hypotonia	hypotonia	mild hypertonia	mild hypertonia	normal	normal	normal
	Reflexes	present	present	present	brisk	present	present	present
Skeletal deformities		-	talipes	arachnodactyly, clinodactyly, clasped thumb	arachnodactyly, clinodactyly, clasped thumb	arachnodactyly	arachnodactyly	arachnodactyly
Behavioral changes		nervousness, sleepless, autistic features	nervousness, autistic features	autistic features, hyperactivity, severe anxiety increasing with age, sleepless	autistic features, hyperactivity	autistic features, hyperactivity, repetitive movements	autistic features, nervousness	few repetitive movements, nervousness
Neuroimaging		thin corpus callosum	thin corpus callosum	thin corpus callosum	thin corpus callosum	thin corpus callosum	thin corpus callosum	thin corpus callosum
Others		drooling, unsteady gait	drooling, startle response to loud sounds, suckling mouth	_	_	_	_	_
Genomic position (hg 38)		chr4-112,647,781 TAAAG > T		chr4-112,647,551 T > C		chr4-112,646,921 T > C		
cDNA alteration		NM_016648.4:c.1113_1116del		NM_016648.4:c.997 + 2 T > C		NM_016648.4:c.518 T > C		
Protein alteration		p.(Glu372Leufs*4)		p.(?)		p.(Phe173Ser)		
gnomAD (v4.1.0)		Not found		0.000001261		6.219e-7		
ACMG Classification (Criteria)		Likely pathogenic (PVS1, PM2)		Likely pathogenic (PVS1, PM2)		VUS (PM2, PP3)

### Immunological profiles

P2, P3, and P5 developed recurrent ear and skin or respiratory infections. Initial immunological evaluation using the ten warning signs of primary immunodeficiency indicated three, four, and six positive signs in P5, P2, and P3, respectively. Their female siblings (P1, P4, P6, and P7) showed no warning signs or only one **(**Supplementary Table 1**)**. Absolute lymphocyte counts and subset counts were within normal ranges, except for mild alterations in cytotoxic T-cell counts in P1, P2, and P3. Serum immunoglobulin levels were normal in all patients **(**Supplementary Table 2**)**. Vaccination history for P1 and P2 confirmed that neither had previously received any type of pneumococcal vaccine, and baseline serological testing for pneumococcal antibodies was negative. Both patients were administered a single dose of the 23-valent polysaccharide vaccine (Pneumovax), and antibody testing was repeated one month later. P1 developed a positive antibody response, whereas her male sibling P2, failed to mount a detectable response. These findings suggest impaired polysaccharide-specific antibody production in P2, consistent with a functional B-cell defect.

### Molecular results

**Fig. 2:**
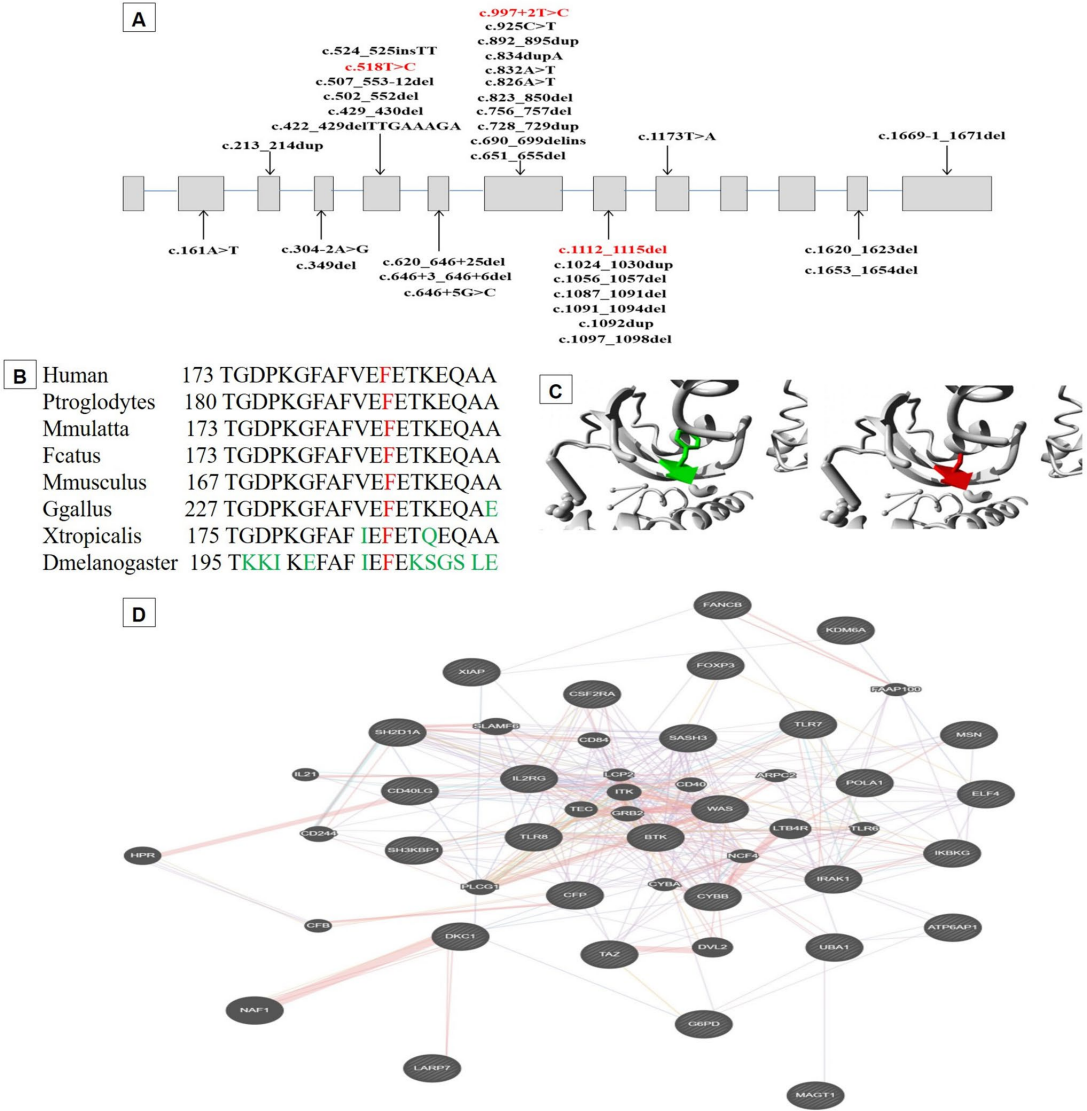
**A**) Spectrum of the disease-causing variants in the *LARP7* gene. Coding exons (boxes) are proportional to with their size. Introns (lines) are not scaled. Variants reported in the study are red-colored. LAM: 28–111, RRM1: 120–188, RRM2: 450–545. **B**) Evolutionary conservation of the Phe173 residue across vertebrate and invertebrate orthologs. Data retrieved from MutationTaster2025 (https://www.genecascade.org/MutationTaster2025/). The target amino acids are shown in red, while not conserved residues are shown in green. **C**) The three-dimensional model of the protein with a close-up of the variant Phe173Ser. The side chains of wild-type and mutant residues are colored green and red, respectively. The protein model was generated using HOPE (https://www3.cmbi.umcn.nl/hope/). Protein sequence and annotation data were obtained from the UniProt database. Secondary structure was predicted using the Reprof software and the three-dimensional structure was analyzed using WHAT IF web services. **D**) Functional protein association between *LARP7* and X-linked immune-related genes was assessed using GeneMANIA (https://genemania.org/)

Three homozygous variants were reported in the study cohort **(**Fig. 2A**)**. The first variant (c.1113_1116del) is a likely pathogenic deletion of 4 nucleotides in exon 8. It changes the reading frame from codon 372, resulting in protein truncation at the 4th codon downstream. The second alteration (c.997 +  2 T > C) is a likely pathogenic splice-site variant, where exon 7 is predicted to be severely affected (SpliceAl: 0.99). The third variant (c.518T > C) is missense resulting in the substitution of a phenylalanine residue at codon 173 with a serine residue (p.Phe173Ser). Phe173 is highly conserved across vertebrate and invertebrate *LARP7* orthologues **(**Fig. 2B**)**. The variant has been predicted to be deleterious by almost all pathogenicity classifiers with potentially damaging effects (Revel = 0.73/MetaRNN = 0.97/CADD = 29.2). It introduces a larger and more hydrophobic amino acid in the RRM1 domain, which is important for protein binding, most likely disturbing its function **(**Fig. 2C**)**. Functional protein association analysis revealed that LARP7 is co-expressed and functionally interacts with the dyskerin gene (DKC1) **(**Fig. 2D**)**.

## Discussion

In 2012, loss of-function variants in *LARP7* were defined as the underlying cause of Alazami syndrome [2]. However, *LARP7* is a relatively small gene consisting of only 13 exons, all patients were molecularly diagnosed by ES, highlighting the disease’s rare incidence and its clinical overlap with other neurodevelopmental disorders. The disease hallmarks are severe intellectual disability, and pre-and postnatal short stature, ranging from primordial dwarfism (height below −3.5 SD) to a less severe phenotype with milder growth restriction (height below −2.5 SD), and distinct dysmorphic facial features including triangular face with prominent forehead, malar hypoplasia, deep-set eyes, low-set ears, short philtrum, and broad nose. Although a triangular facial shape has been described as a characteristic feature in patients with Alazami syndrome, some of our patients (*n* = 4) exhibited a long facial appearance instead. However, the overall long face in such patients may be attributable to other morphological characteristics, such as a high forehead, midface hypoplasia, and a pointed chin that can elongate the facial profile.

Although an underdeveloped maxilla has been reported in Alazami syndrome [16], our patients exhibited a prominent premaxilla, with six presenting anterior open bite. P7 showed retrognathia, consistent with findings by Kazemi et al. (2020) [17]. Midface hypoplasia and high-arched palate, characteristic features of the syndrome [16, 18], were also observed in our cohort, except for P3 who had shallow palate and P7 who presented without malar hypoplasia. Short philtrum and macrostomia, previously described by Imbert-Bouteille et al. (2019) [19], were similarly noted in our patients, except in P5, who showed a long, broad philtrum, and in P5–P7, who had normal mouth width. All examined patients had thin lips, consistent with Wojcik et al. (2019) [20], whereas full lips were more frequently reported by Imbert-Bouteille et al. (2019) [19]. Widely spaced and malposed teeth, features previously associated with Alazami syndrome [19, 21], were evident in P3 and P5, possibly contributing to maxillary growth disturbances. Finally, enamel structure defects were observed in most patients; however, the exact role of LARP7 in this phenotype remains unclear.

As a neurodevelopmental disorder, patients with Alazami syndrome presented a wide range of neurological manifestations extending beyond mental retardation and speech impairment to include seizures [3, 17, 22, 23], epilepsy [3, 11, 22, 23] and behavioral changes such as anxiety [19, 24–26], aggression [10], hyperactivity [24] and hypersensitivity [25]. There is no document for brain MRI in several patients with Alazami syndrome. Among the reported MRIs, no lesion has been found in some patients [2, 3]. In the current study, a thin corpus callosum has been reported in all patients. The same finding has been reported in a previous study [20]. Other formerly described brain malformations in disease patients included unilateral mild insular and anterior frontal gyrus cortical thickening [2], increased signal in periventricular white matter [17], corpus callosum agenesis, absence of septum pellucidum [4], disorganized cerebellar hemispheres [20], Arnold Chiari malformation type 1, venous angioma [22], and mild myelination delay [27].

Other complications, including circulatory, genitourinary, skeletal, and immunological systems, have been reported in some patients. No circulatory or genitourinary defects were evident among the study participants. However cardiac abnormalities in the form of arterial septal defect [3, 5, 12, 17, 19] and pulmonary stenosis [4, 22] were previously indicated. Nephropathic changes such as renal artery stenosis [3], ureteric stones [3], and obstruction of the ureteropelvic junction [5] were described in the disease patients. Congenital anomalies of the male genital tract were mainly represented by undescended testes [2, 3, 12], micropenis [3], and hypospadias [4, 28]. Skeletal abnormalities such as arachnodactyly, clinodactyly, clasped thumb, and talipes were detected in our patients. However scoliosis and kyphoscoliosis seem to be frequent among patients with Alazami syndrome [2, 4, 5, 17, 19, 24], they were not observed in the study subjects. Immunological defects were rarely described in patients with Alazami syndrome. Recurrent otitis with upper respiratory tract infections [23] and impaired antibody responses to pneumococcal vaccination [28] were reported in only two male patients. In the current study, the three male patients developed recurrent infections and met ≥ 2 warning signs of primary immunodeficiency. The immunological profile of the patients suggests that humoral immunodeficiency may be a feature of this syndrome. Additional immunological studies, including extended B-cell phenotyping, may provide further insights into the spectrum of humoral immune involvement associated with *LARP7* deficiency. The observed sex-related differences in humoral immune findings raise the possibility of modifying factors influencing immune expression in Alazami syndrome. Bioinformatic network analysis suggested potential associations between LARP7 and pathways involved in immune regulation [15]. Any potential relationship between LARP7 function, immune regulation, and sex-specific modifiers require dedicated experimental validation and confirmation in independent cohorts.

The gene appears to contain a hotspot mutational region spanning exons 5 to 8, which harbors about 80% of the identified gene variants **(**Fig. 2A**)**. The reported variants are predominantly frameshift, nonsense, and splice-site alterations, suggesting that the disease primarily results from a loss-of-function mechanism. The first missense gene variant was recently reported in 2025 [29], and the second one was identified in the current study, expanding the mutational spectrum of the gene. The majority of patients with Alazami syndrome, including those in our cohort, were born to consanguineous parents and carried homozygous variants. Compound heterozygous variants were reported in only four families [5, 20, 23, 30]. Generally, each variant is reported in a single family, underscoring the high mutational variability of the gene. However, some variants have been reported in more than one family from the same ethnicity, including c.646 + 3_646 + 6del, c.1024_1030dup, c.1173T > A and c.1653_1654delAA [2, 3, 24]. The variant c.651_655del was also detected in two families from the USA [25] and Canada [29]. Moreover, the variant c.834dupA was reported in two families from the USA and Spain in a compound heterozygous state with c.646 + 5G > C [20] and NM_016648.4:c.690_699delins27 [23], respectively. Current evidence suggests that there is not a clear or robust genotype–phenotype correlation in Alazami syndrome. Although all affected individuals share the core clinical hallmarks of short stature or growth restriction, intellectual disability, and distinctive facial features, the severity and additional manifestations vary, even among patients with similar variant types, and there is no consistent pattern that links specific LARP7 genotypes with distinct clinical sub‑phenotypes. Further studies with larger cohorts and systematic genotype and phenotype characterization are needed to determine whether specific LARP7 variants correlate with particular clinical features or severity, and to clarify the range of expressivity within and between families.

## Conclusion

The first delineation and subsequent characterization of Alazami syndrome have been granted by the high throughput technology of next-generation sequencing. The low incidence, clinical overlapping, and heterogeneous phenotype of the syndrome pose considerable challenges in the realms of diagnosis and management. Prominent premaxilla and enamel structure defects observed as oro-dental phenotypes in the study cohort represent new findings that have not been previously reported. Our immunological findings suggest that immunodeficiency may be an underrecognized feature of Alazami syndrome, potentially with male sex predilection, warranting systematic immunological evaluation in future cases. An accurate and thorough description of the disease manifestations and complications would boost our understanding of the condition’s mechanisms and pathways.

## Supplementary information

Below is the link to the electronic supplementary material.

Supplementary file1 (JPG 36 KB)

Supplementary file2 (DOCX 14 KB)

Supplementary file3 (DOCX 19 KB)

## Data Availability

The datasets supporting the findings of this article are included within the article and its supplementary files.
