# Supplementary figures and images for: Expanding the phenotypic and immunological landscape of Alazami syndrome: Evidence from seven new patients with LARP7 gene variants
Source: Eur J Pediatr. 2026 Mar 11;185(4):175. doi: 10.1007/s00431-026-06801-0 (PMC12979316; doi:10.1007/s00431-026-06801-0)

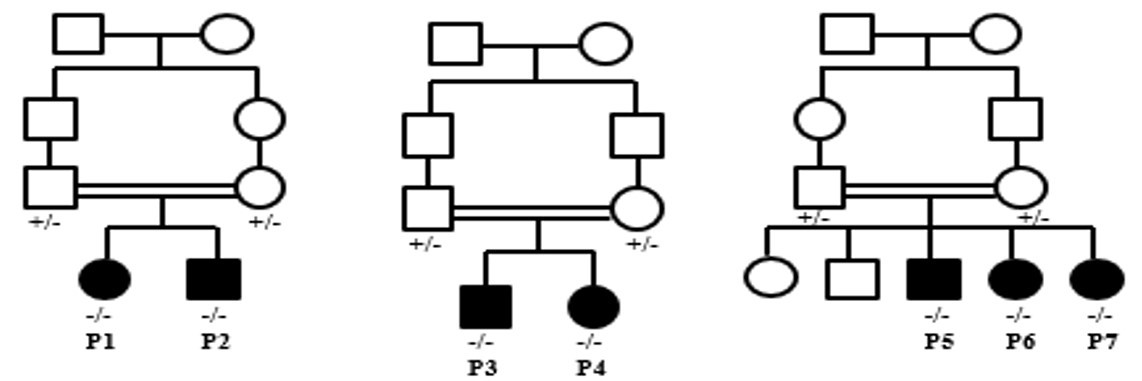

Supplement: Supplementary file 1 — Supplementary file1 (JPG 36 KB) [file 431_2026_6801_MOESM1_ESM.jpg]
