# Supplementary material for: Expanding the phenotypic and immunological landscape of Alazami syndrome: Evidence from seven new patients with LARP7 gene variants
Source: Eur J Pediatr. 2026 Mar 11;185(4):175. doi: 10.1007/s00431-026-06801-0 (PMC12979316; doi:10.1007/s00431-026-06801-0)
Supplement: Supplementary file 2 — Supplementary file2 (DOCX 14 KB) [file 431_2026_6801_MOESM2_ESM.docx]

**Supplementary Table 1:** warning signs of primary immunodeficiency among study participants

| **P1** | **P2** | **P3** | P4 | **P5** | **P6** | **P7** |
| --- | --- | --- | --- | --- | --- | --- |
| None | 1. Four or more new ear infections within one year  2. Two or more pneumonias within one year  3. Failure of an infant to gain weight or grow normally  4. Need for intravenous antibiotics to clear infections | 1. Four or more new ear infections within one year  2. Two or more months on antibiotics with little effect  3. Two or more pneumonias within one year  4. Failure of an infant to gain weight or grow normally  5. Recurrent, deep skin or organ abscesses  6. Need for intravenous antibiotics to clear infections | 1. Two or more months on antibiotics with little effect | 1. Four or more new ear infections within one year  2. Two or more serious sinus infections within one year  3. Persistent thrush in mouth or fungal infection on skin | None | None |
