# Supplementary material for: Expanding the phenotypic and immunological landscape of Alazami syndrome: Evidence from seven new patients with LARP7 gene variants
Source: Eur J Pediatr. 2026 Mar 11;185(4):175. doi: 10.1007/s00431-026-06801-0 (PMC12979316; doi:10.1007/s00431-026-06801-0)
Supplement: Supplementary file 3 — Supplementary file3 (DOCX 19 KB) [file 431_2026_6801_MOESM3_ESM.docx]

**Supplementary Table 2:** Immunological profiles of the studied patients

| **Parameter** | **P1** | **P2** | **P3** | P4 |
| --- | --- | --- | --- | --- |
|  |  |  |  |  |
| - **RBCs** | N | N | N | Mild microcytic hypochromic anaemia |
| - **Hb (g/dl)** | 12.2 | 12.2 | 13.3 | 11.4 |
| - **WBCs** | N | N | N | N |
| - **Platelets** | N | N | N | N |
| **Cellular immunity** |  |  |  |  |
| - **CD3 T lymphocytes** | N | N | N | N |
| **Relative count, %** | 62 (RR 56-75) | 63.1 (RR 56-75) | 81 (RR 52-83) | 69 (RR 56-84) |
| **Absolute count, cells/μl** | 2804 (RR 1400-3700) | 2991 (RR 1400-3700) | 1952 (RR 605-2921) | 1773 (RR 1000-2200) |
| - **CD4 T helper** | N | N | N | N |
| **Relative count, %** | 28 (RR 28-47) | 31 (RR 28-47) | 37.8 (RR 24-60) | 47.5 (RR 31-52) |
| **Absolute count, cells/μl** | 1256 (RR 700-2200) | 1469 (RR 700-2200) | 911 (RR 360-1600) | 1220 (RR 530-1300) |
| - **CD8 T cytotoxic** | Mild decrease in relative count | Decreased relative & absolute count | Mild increase in absolute count | N |
| **Relative count, %** | 14.5 (RR 16-30) | 9.6 (RR 16-30) | 32.2 (RR 14-35) | 22 (RR 18-35) |
| **Absolute count, cells/μl** | 655 (RR 490-1400) | 455 (RR 490-1400) | 776 (RR 170-755) | 565 (RR 330-920) |
| - **CD4/CD8 Ratio** | N  1.93 (RR 1.26–2.9) | Elevated  3.2 (RR 1.26–2.9) | N  1.17 (RR 1.06–2.76) | N  2.15 (RR 1.21–2.64) |
| - **CD19 B lymphocytes** | N | N | N | N |
| **Relative count, %** | 22.3 (RR 14-33) | 23.5 (RR 14-33) | 8.1 (RR 3.5-25) | 13.4 (RR 6-23) |
| **Absolute count, cells/μl** | 1008 (RR 390-1400) | 1114 (RR 390-1400) | 195 (RR 107-752) | 344 (RR 110-570) |
| - **CD16 NK cells** | N | N | N | N |
| **Relative count, %** | 5 (RR 4-17) | 4 (RR 4-17) | 5.1 (RR 3-22) | 6.3 (RR 3-22) |
| **Absolute count, cells/μl** | 208 (RR 130-720) | 190 (RR 130-720) | 123 (RR 70-480) | 162 (RR 70-480) |
| **Humoral Immunity**  **IgG**  **IgM**  **IgA** | Normal IgG  Normal IgM  Normal IgA | Normal IgG  Normal IgM  Normal IgA | Normal IgG  Normal IgM  Normal IgA | Normal IgG  Normal IgM  Normal IgA |
